# Hip Fracture Incidence in Relation to Age, Menopausal Status, and Age at Menopause: Prospective Analysis

**DOI:** 10.1371/journal.pmed.1000181

**Published:** 2009-11-10

**Authors:** Emily Banks, Gillian K. Reeves, Valerie Beral, Angela Balkwill, Bette Liu, Andrew Roddam, for the Million Women Study Collaborators

**Affiliations:** 1 National Centre for Epidemiology and Population Health, The Australian National University, Acton, Australia; 2 Cancer Research UK Epidemiology Unit, University of Oxford, Oxford, United Kingdom; UKK Institute, Finland

## Abstract

**Background:**

Bone mineral density is known to decrease rapidly after the menopause. There is limited evidence about the separate contributions of a woman's age, menopausal status and age at menopause to the incidence of hip fracture.

**Methods and Findings:**

Over one million middle-aged women joined the UK Million Women Study in 1996–2001 providing information on their menopausal status, age at menopause, and other factors, which was updated, where possible, 3 y later. All women were registered with the UK National Health Service (NHS) and were routinely linked to information on cause-specific admissions to NHS hospitals. 561,609 women who had never used hormone replacement therapy and who provided complete information on menopausal variables (at baseline 25% were pre/perimenopausal and 75% postmenopausal) were followed up for a total of 3.4 million woman-years (an average 6.2 y per woman). During follow-up 1,676 (0.3%) were admitted to hospital with a first incident hip fracture. Among women aged 50–54 y the relative risk (RR) of hip fracture risk was significantly higher in postmenopausal than premenopausal women (adjusted RR 2.22, 95% confidence interval [CI] 1.22–4.04; *p = *0.009); there were too few premenopausal women aged 55 y and over for valid comparisons. Among postmenopausal women, hip fracture incidence increased steeply with age (*p<*0.001), with rates being about seven times higher at age 70–74 y than at 50–54 y (incidence rates of 0.82 versus 0.11 per 100 women over 5 y). Among postmenopausal women of a given age there was no significant difference in hip fracture incidence between women whose menopause was due to bilateral oophorectomy compared to a natural menopause (adjusted RR 1.20, 95% CI 0.94–1.55; *p = *0.15), and age at menopause had little, if any, effect on hip fracture incidence.

**Conclusions:**

At around the time of the menopause, hip fracture incidence is about twice as high in postmenopausal than in premenopausal women, but this effect is short lived. Among postmenopausal women, age is by far the main determinant of hip fracture incidence and, for women of a given age, their age at menopause has, at most, a weak additional effect.

*Please see later in the article for the Editors' Summary*

## Abstract 2

Using data from the UK Million Women Study, Emily Banks and colleagues investigate the relationships between the incidence of hip fracture and a woman's age, menopausal status, and age at menopause.

## Editors' Summary

**Background:**

Anyone can break a hip but most hip fractures occur in elderly people. As people age, their bones gradually lose minerals and become less dense, which weakens the bones and makes them more susceptible to fracture. Because women lose bone density faster than men as they age and because women constitute the majority of the elderly, three-quarters of hip fractures occur in women. Hip fractures can cause long-term health problems and premature death. Thus, although surgical repair of a broken hip usually only requires a hospital stay of about a week, a quarter of elderly people who were living independently before their fracture have to stay in a nursing home for at least a year after their injury and a fifth of elderly people who break a hip die within the year. Most hip fractures are caused by falls. Regular exercise to improve strength and balance combined with review of medicines (to reduce side effects and interactions), regular eye examinations, and the removal of fall hazards from the home can help to prevent hip fractures in elderly people.

**Why Was This Study Done?:**

Bone density decreases very rapidly in women immediately after menopause—the time when menstruation permanently stops—and then continues to decrease more slowly with age. Most women have their menopause in their early 50s but menopause can occur in younger women. Early menopause is thought to be a risk factor for osteoporosis (thinning of the bones) and fractures later in life but little is known about how menopause influences hip fracture risk as women age. In this prospective study (a type of study in which a group of people is followed for several years to see whether they develop a particular condition), the researchers investigate the incidence of hip fractures in relation to age, menopausal status, and age at menopause among the participants of the Million Women Study. This study, which recruited 1.3 million women aged 50–64 years who attended UK breast cancer screening clinics between 1996 and 2001, has been investigating how reproductive and lifestyle factors affect women's health.

**What Did the Researchers Do and Find?:**

At enrollment and three years later, the study participants provided information about their menopausal status and other health and lifestyle factors likely to affect their fracture risk. From these data, the researchers identified more than half a million women who had never used hormone replacement therapy (which reduces fracture risk) and who had given complete information about their menopausal status. They then looked for statistical associations between the occurrence of a first hip fracture in these women over the next few years and their age, menopausal status, and age at menopause. Among women aged 50–54 years, postmenopausal women were twice as likely to have a hip fracture as premenopausal women. Among postmenopausal women, the incidence of hip fractures increased steeply with age and was seven times higher in 70–74-year olds than in 50–54-year olds. Women who had their menopause before age 45 had a slightly increased risk of hip fracture but any effect of age at menopause on the risk of hip fracture was small compared to the effect of age itself, and the slightly increased risk may have been due to other factors that could not be fully accounted for in the analysis.

**What Do These Findings Mean?:**

These findings indicate that around the time of menopause, although hip fractures are rare, the risk of a fracture in postmenopausal women is twice that in premenopausal women. The findings also show that among postmenopausal women, age is the major determinant of hip fracture risk and that for women of a given age, their age at menopause has little effect on hip fracture risk. Women attending breast cancer screening clinics and completing questionnaires about their health may not be representative of the general population. Furthermore, these findings rely on women self-reporting their menopausal status accurately. Nevertheless, the results of this study suggest that clinicians advising women about hip fracture prevention should probably base their advice on the woman's age and on age-related factors such as frailty rather than on factors related to menopause. Clinicians can also now reassure elderly women who had an early menopause that their risk of hip fracture is unlikely to be higher than that of similar women who had a later menopause.

**Additional Information:**

Please access these Web sites via the online version of this summary at http://dx.doi.org/10.1371/journal.pmed.1000181.

- The American Academy of Orthopaedic Surgeons has detailed information about hip fractures
- The US National Institute of Arthritis and Muscoloskeletal and Skin Diseases has an interactive feature called “Check up on your bones and provides detailed information about osteoporosis, including advice on fall prevention
- The US Centers for Disease Control and Prevention has a fact sheet about hip fractures among older adults
- MedlinePlus has links to resources about hip fracture, osteoporosis, and menopause (in English and Spanish)
- More information on the Million Women Study is available

## Introduction

As women age, hip fractures become an increasingly important cause of morbidity and mortality. Reduced bone mineral density is a key risk factor for hip fracture [1]. Bone density is known to decrease rapidly in the period immediately after the menopause and more slowly thereafter [2]–[4]. Although early menopause is widely considered to be a risk factor for osteoporosis and fracture in later life [5], there is limited evidence about how menopause influences hip fracture risk as women grow older, and uncertainty around whether an earlier menopause leads to a lasting increase in risk [6],[7]. Previous studies have generally not found a significant effect of younger age at menopause on hip fracture risk [6],[8]–[19], although many of these studies were small and had limited ability to account for potential confounding factors. This study describes the incidence of hip fracture in relation to age, menopausal status, and age at menopause in a large cohort of UK women who have never used hormone replacement therapy.

## Materials and Methods

### Ethics Statement

The study has been approved by the Eastern Multi-centre Research Ethics Committee and all study participants provided written consent to take part.

### Data Collection and Definitions

The Million Women Study is a large national prospective study that recruited 1.3 million middle-aged women from National Health Service (NHS) breast screening clinics in England and Scotland in 1996–2001; its methods are described elsewhere [20]. Participants provided information on sociodemographic and anthropometric factors, menopausal status, use of hormone replacement therapy, and medical and reproductive history by filling in a questionnaire at recruitment. The study population was resurveyed in 1999–2004, about 3 y after recruitment, with a response rate of 65%. Study questionnaires can be viewed at www.millionwomenstudy.org.uk.

All study participants were registered with the NHS at recruitment. Their unique NHS personal identifying number is used for all NHS health records, including those relating to the breast cancer screening programme and hospital admissions. All study participants have been linked electronically using their unique NHS number and other personal details, to obtain information on NHS hospital admissions, cancer registrations, deaths, and emigrations. The hospital data include details of hospital admissions in study participants in Scotland from 1st January 1981 to the 31st of December 2003 [21] and in England from 1st April 1997 to the 31st of March 2005 [22]. For every hospital episode the date of admission is recorded, as is the primary reason for admission, with up to 13 additional clinical diagnoses coded using the World Health Organisation International Classification of Diseases 9th and 10th revisions (ICD-9 and ICD-10) [23]. As well, up to 12 operations or procedures are coded for each hospital episode using the Office of Population Censuses and Surveys Classification of Surgical Operations and Procedures, 3rd and 4th revisions (OPCS-3 and OPCS-4) [24].

### Analysis

For these analyses the main outcome, hip fracture, is defined as the first hospitalisation following recruitment into the Million Women Study with a final primary diagnosis of fractured neck of femur at discharge (ICD-10 codes S72.0–S72.2 and S72.9). Analyses are restricted to women reporting at recruitment that they had never used hormone replacement therapy, since use of hormone replacement therapy is closely related to a woman's menopausal status and her age at menopause [25] and has a substantial impact on the risk of fracture [26],[27]. Of the 659,737 women reporting at recruitment that they had never used hormone replacement therapy, 25,218 (3.8%) who had a cancer registered prior to recruitment, other than nonmelanoma skin cancer (ICD10 C44), were excluded because a substantial proportion of cancers in women, and their treatments, relate to hormonal factors and a previous diagnosis of cancer is likely to influence the risk of hip fracture. A further 68,551 women who had an unknown age at menopause because of having had a hysterectomy without bilateral oophorectomy prior to natural menopause, and 4,359 who were missing data on menopausal status, were also excluded leaving 561,609 women for the main analyses.

Woman-years were calculated from the date of recruitment up to the date of first hospital admission for the hip fracture, date of death, date of emigration, or the end of follow-up, whichever came first. For women recruited in Scotland the last date of follow-up was the 31st of December 2003 and for those in England the last date was the 31st of March 2005, corresponding to the dates after which the hospital records were still incomplete at the time of record-linkage. Woman-years were calculated from 1st April 1997 for the small proportion of women (5%) recruited in England before that date, as hospital admission data were not available before then. Menopausal status and age at menopause were classified initially using information provided at recruitment, and were reclassified whenever possible using updated information on menopause reported at resurvey, as described previously [28],[29]. For 226 (13%) out of 1,676 women with a first incident hip fracture, self-reported information on the cause of the hip fracture was available from the follow-up questionnaire.

Premenopausal women were defined as those who reported having regular menstrual periods, perimenopausal women were those who reported that their periods were irregular because of the menopause, and postmenopausal women were those reporting cessation of their periods, as the result of natural menopause or a bilateral oophorectomy. These baseline measures were checked against follow-up data obtained approximately 3 y later. Of 47,859 women classified as perimenopausal at recruitment, 19.6% (9,386) were perimenopausal and 77.5% (37,066) were postmenopausal at follow-up. In contrast, of 40,495 women who were classified as premenopausal at recruitment, 28.2% (11,423) were premenopausal, 32.9% (13,337) were perimenopausal, and 38.9% (15,735) were postmenopausal at follow-up. This finding suggests that our definitions are predictive of the timing of the menopausal transition. Data from premenopausal and perimenopausal women were censored 48 mo after baseline if they reported being pre- or perimenopausal at follow-up or did not return a follow-up form, as their status is likely to have altered subsequently.

Relative risks (RRs) for hip fracture according to menopausal status and age at menopause were estimated using a Cox regression model, in which the underlying time variable was the woman's age. RRs and 95% confidence intervals (CIs) take account of age (the underlying time variable) and region and were adjusted further for: socioeconomic status (in quintiles, measured by Townsend score, which is based on car and home ownership, overcrowding, and unemployment in the area of residence [30]); body mass index (<22.5, 22.5–24.9, 25.0–27.4, 27.5–29.9, ≥30 kg/m^2^); cigarette smoking (current, past, never); alcohol consumption (never, <1, ≥1 drinks per week); strenuous physical activity (<1, ≥1 time per week); parity (nulliparous, parous); previous use of oral contraceptives (ever, never); and medical history (history of heart disease/stroke/thrombosis, diabetes mellitus, thyroid disease, rheumatoid arthritis, and osteoarthritis). Results were unchanged following adjustment for finer categories of alcohol consumption and strenuous physical activity; the broader categories were therefore retained. The RR of hip fracture in postmenopausal women who had menopause resulting from a bilateral oophorectomy compared to those with a natural menopause was adjusted for all of the factors mentioned above, as well as age at menopause. The proportional hazards assumption was tested using Schoenfeld residuals. Sensitivity analyses were conducted examining the effect on the exposure of interest of stratifying by adjustment variables showing nonproportionality and of excluding women with a hip fracture recorded prior to recruitment. The STATA 10.1 computing package was used for all analyses [31].

## Results

The characteristics of the study population are presented in Table 1 according to menopausal status and age at menopause, at recruitment. Of the 561,609 never users of hormone replacement therapy included in the analyses, 25% were pre/perimenopausal and 75% were postmenopausal at recruitment and, as expected, pre/perimenopausal women at recruitment were younger than the postmenopausal women (mean age 51 y versus 58 y). Various differences in the characteristics of the pre/perimenopausal and postmenopausal women largely reflect their younger age. Among the postmenopausal women 11% reported that their menopause had occurred before age 45 and 61% reported that their menopause had occurred at age 50 y or older. Women with an early menopause (before age 45 y) differed substantially from women with a later menopause (at 50 y or later)—as well as being more likely to have had a bilateral oophorectomy they were more likely to be of lower socioeconomic status, more likely to smoke, and more likely to have a history of heart disease, stroke, thrombosis, diabetes, thyroid disease, and arthritis (*p<*0.001).

**Table 1:** Characteristics of study population (never users of hormone replacement therapy) according to menopausal status at recruitment and age at menopause.

Study Population	Pre/Perimenopausal	Age (y) at Menopause among Postmenopausal Women			All Postmenopausal
		<45	45–49	50+	
**Number of women**	141,886	49,132	124,342	233,505	419,723
**Characteristics at recruitment**					
**Age (mean [SD], y)**	51.2 [1.9]	58.4 [4.6]	57.1 [4.9]	58.5 [4.2]	58.1 [4.5]
**Bilateral oophorectomy (% [** ***n*** **])**	0% [0]	11% [5,334]	2% [2,938]	1% [2,538]	3% [11,299]
**Socioeconomic status (% [** ***n*** **] in lower third)**	29% [40,970]	43% [21,032]	37% [45,873]	32% [73,962]	35% [145,950]
**Parity (% [** ***n*** **] with parity ≥2)**	75% [106,382]	69% [33,889]	71% [88,779]	74% [173,904]	73% [305,269]
**Past use of oral contraceptives (% [** ***n*** **])**	69% [98,508]	43% [21,358]	48% [59,709]	42% [98,292]	44% [184,034]
**Current smoker (% [** ***n*** **])**	14% [19,333]	29% [14,364]	24% [29,330]	14% [33,191]	19% [79,413]
**Alcohol consumption (mean [SD], g/week)**	46.5 [55.4]	32.2 [48.6]	35.6 [50.0]	35.9 [49.2]	35.2 [49.4]
**Body-mass index (mean [SD], kg/m^2^)**	26.2 [5.0]	26.5 [5.1]	26.2 [4.8]	26.5 [4.8]	26.4 [4.9]
**Strenuous physical activity (% [** ***n*** **] more than once/week)**	41% [57,933]	31% [15,002]	35% [43,333]	38% [88,848]	36% [151,062]
**History of heart disease, stroke or thrombosis (% [** ***n*** **])**	5% [7,496]	15% [7,150]	10% [12,525]	10% [22,569]	10% [43,796]
**History of diabetes mellitus (% [** ***n*** **])**	2% [2,631]	4% [2,175]	3% [4,024]	3% [8,017]	4% [14,884]
**History of thyroid disease (% [** ***n*** **])**	4% [6,065]	7% [3,480]	6% [7,383]	6% [13,541]	6% [25,272]
**History of rheumatoid arthritis (% [** ***n*** **])**	2% [3,040]	7% [3,507]	5% [6,064]	4% [9,713]	5% [20,092]
**History of osteoarthritis (% [** ***n*** **])**	4% [5,375]	10% [4,727]	7% [9,266]	7% [17,428]	8% [32,450]
**Follow-up for hip fracture**					
**Woman-years of follow-up**	769,522	297,985	763,512	1,441,796	2,581,140
**First hospital admission for hip fracture (** ***n*** **)**	86	252	464	801	1590

Women with missing values are not included. SD, standard deviation.

The study population was followed for a total of 3,350,662 woman-years; an average of 6.2 y per woman. During follow-up 1,676 (0.3%) women were admitted to hospital with a first incident hip fracture, on average, 3.7 y after recruitment. Of the women with a hip fracture who had self-reported data on the cause of the hip fracture, 88% (199/226) were the result of a fall, 1% (2/226) the result of a motor vehicle accident, 1% (3/226) due to some other accident, 5% (11/226) occurred in some other way, and 5% (11/226) did not specify a cause.

### Menopausal Status

**Figure 1:**
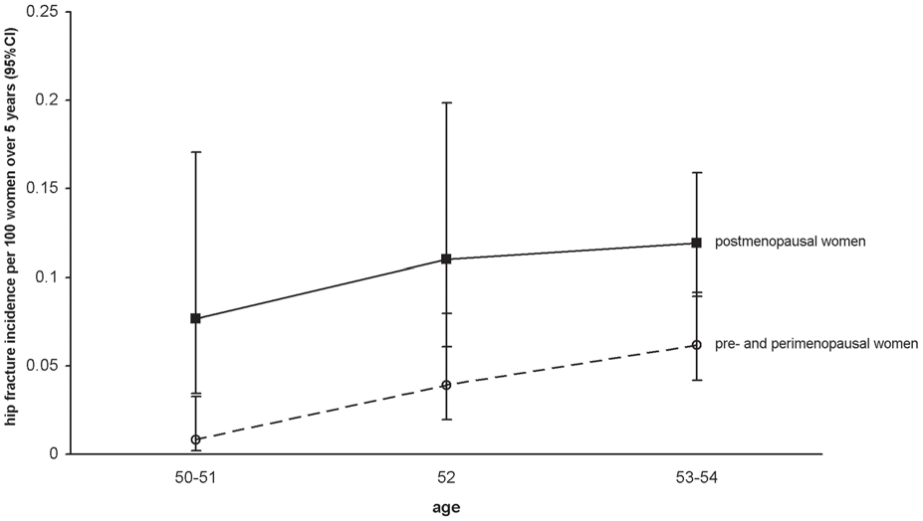
Age-specific incidence of hip fracture in the study population who have never used hormone replacement therapy, according to menopausal status.

Since most women become menopausal in their late 40s and early 50s, there is limited scope for comparing the incidence of hip fracture by menopausal status in women of a given age. So that we could directly examine the effect of menopausal status in groups of women of similar age we restricted analyses to women within a narrow age range from 50 to 54 y. In these analyses participants contributed woman-years only up to the day they turned 55, and were censored thereafter. For women aged 50–54 y, the RR of hip fracture in postmenopausal women was more than double that in premenopausal women of a similar age (adjusted RR 2.22, 95% CI 1.22–4.04, *p = *0.009; Table 2). Excluding the 42 women aged 50–54 y with a hip fracture recorded prior to recruitment into the study, findings were unchanged (adjusted RR 2.22 [1.22–4.04] in premenopausal women, compared to postmenopausal women). The risk of hip fracture in premenopausal and perimenopausal women aged 50–54 y did not differ significantly (Table 2), however the relatively wide CI means that a substantial increase in the RR of hip fracture in perimenopausal compared to premenopausal women cannot be excluded reliably. Figure 1 shows the age-specific incidence of hip fracture for pre/perimenopausal women and postmenopausal women.

**Table 2:** Hip fracture incidence and RR of hip fracture by menopausal status among women aged 50–54 y who never used hormone replacement therapy.

Menopausal Status	Hip Fracture Incidence per 100 Women over 5 y [*n*]	RR^a^	RR^b^ (95% CI)
**Premenopausal**	0.03 [14]	1.00	1.00
**Perimenopausal**	0.05 [21]	1.30	1.25 (0.63–2.46)
**Postmenopausal**	0.11 [63]	2.83	2.22 (1.22–4.04)

Data are censored at age 55.

a Adjusted for age and region only.

b Adjusted for age, region, socioeconomic status, body-mass index, cigarette smoking, alcohol consumption, physical activity, oral contraceptive use, parity, and medical history.

### Effect of Age among Postmenopausal Women

**Figure 2:**
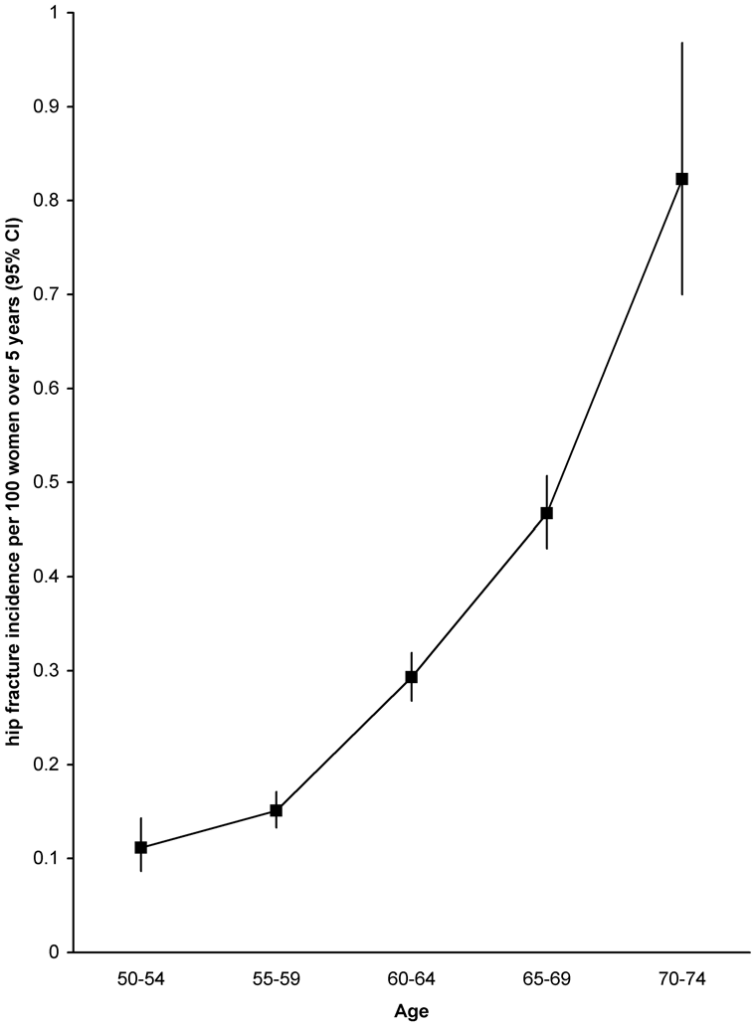
Age-specific incidence of hip fracture in postmenopausal women in the study population who have never used hormone replacement therapy.

Among postmenopausal women, the incidence of hip fracture increased substantially with age, such that incidence rates were about seven times higher at age 70–74 than 50–54 (Figure 2). Incidence rates per 100 women over 5 y were 0.11, 0.15, 0.29, 0.48, and 0.82, respectively, at ages 50–54, 55–59, 60–64, 65–69, and 70–74 (test for trend, *p<*0.001).

### Type of and Age at Menopause among Postmenopausal Women

Among postmenopausal women, the RR of hip fracture did not vary significantly according to whether menopause occurred naturally or as the result of a bilateral oophorectomy (adjusted RR for bilateral oophorectomy versus natural menopause is 1.20, 95% CI 0.94–1.55; *p = *0.15), however this CI is fairly wide and a small differential effect of bilateral oophorectomy on hip fracture cannot be ruled out.

Among postmenopausal women, there is, at most, a weak effect of age at menopause on the risk of hip fracture (Table 3). Adjustment for socioeconomic group, smoking, and other factors, including medical history, substantially reduced the RR estimates that were adjusted by age and region only (from 1.52 to 1.22 for menopause before 45 y, and from 1.23 to 1.08 for menopause at age 45–49 y, compared to 50 y and over, Table 3). The corresponding χ^2^ value declined by 80% or more, from 30.7 to 6.1 for menopause aged <45 y and from 11.8 to 1.6 for menopause at 45–49 y, compared to menopause at ≥50 y. Since most of the factors adjusted for are measured with some error, and adjustment for them reduced the χ^2^ by 80% or more, adjustment for their true values would be expected to reduce the χ^2^ to an even greater extent. This result suggests that there may well be residual confounding in the adjusted estimate.

**Table 3:** Hip fracture incidence and RR of hip fracture among postmenopausal women who have never used hormone replacement therapy, by age and age at menopause.

Age at Menopause (y)	Hip Fracture Rate per 100 Women over 5 y [*n*]					RR^a^	RR^b^ (95% CI)
	50–54 Age Group (y)	55–59 Age Group (y)	60–64 Age Group (y)	65–69 Age Group (y)	70–74 Age Group (y)		
**<45**	0.15 [9]	0.24 [35]	0.39 [82]	0.63 [101]	1.04 [24]	1.52	1.22 (1.05–1.40)
**45**–**49**	0.13 [35]	0.18 [79]	0.33 [152]	0.47 [153]	0.96 [45]	1.23	1.08 (0.97–1.22)
**50+**	0.08 [17]	0.13 [124]	0.26 [281]	0.43 [319]	0.72 [77]	1.00	1.00
**Total**	0.11 [61]	0.15 [238]	0.29 [515]	0.47 [573]	0.82 [146]	—	—

a Adjusted for age and region only.

b Adjusted for age, region, socioeconomic status, body-mass index, cigarette smoking, alcohol consumption, physical activity, oral contraceptive use, parity, and medical history.

The RRs for the effect of menopausal status and age at menopause on hip fracture conformed to the proportional hazards assumption. These RRs changed by <5% following stratification for adjustment variables demonstrating nonproportionality, namely BMI, cardiovascular disease, and arthritis. Hence, the original models were retained.

## Discussion

This study shows that of the factors considered here, the main determinants of the incidence of hip fracture are age and menopausal status. At around the time of menopause, when women are aged 50–54 y, the incidence of hip fracture was significantly higher in postmenopausal than in premenopausal women. There are too few premenopausal women aged 55 y and older to permit valid comparison. Previous publications directly observing the relationship between hip fracture risk in relation to menopause generally involve older, postmenopausal women [6],[9]–[14], and we were unable to locate any publications comparing hip fracture rates between premenopausal and postmenopausal women of a similar age. The rapid and substantial impact of menopause on the risk of hip fracture is likely to relate to the large reductions in bone mineral density that occur in the first few years after the menopause [2]–[4], which follow the rapid reductions in circulating levels of estradiol and related hormones occurring at that time. This finding is in keeping with the established protective effect of both endogenous [32] and exogenous estrogens on fracture incidence [26],[27].

In postmenopausal women the incidence of hip fracture increases rapidly with age, with incidence rates in women aged 70–74 y being about seven times higher than at 50–54 (Figure 2). The fact that hip fracture incidence increases rapidly with age in middle-aged and older women is well established [9],[10],[12].

Our finding of no significant difference in hip fracture incidence between postmenopausal women whose menopause resulted from a bilateral oophorectomy compared to a natural menopause, after adjusting for age and other relevant factors, is consistent with the published findings from other studies [33],[34], however the relatively wide CI means that a small effect on risk remains possible.

We found that women's age at menopause had, at most, a weak effect on the risk of hip fracture in postmenopausal women of a given age. Women who had a menopause before age 45 differ from those with a later menopause in many important respects; they are more likely to have a history of various illnesses as well as being more likely to smoke and to come from the lower socioeconomic groups. Statistical adjustment for medical history, as well as smoking and socioeconomic group, substantially reduced the RR estimates for a menopause before age 45 versus at age 50 or older that were adjusted by age and region only (from 1.5 to 1.2), suggesting that these are important confounding factors. Our findings are in keeping with previous studies that have shown that the vast majority of hip fractures are the result of a fall [35]–[37]. No information was collected on some other risk factors for hip fracture, including previous falls and sensory impairment and there was limited information on previous fractures. This lack of information and the fact that the variables that were adjusted for are measured with some error makes it likely that there is residual confounding in the adjusted estimate.

Despite the widespread belief that early menopause is a long term risk factor for osteoporosis and fracture [5], the published evidence is in keeping with our finding of little, if any, effect of age at menopause on the risk of hip fracture in postmenopausal women, over and above the strong effect of women's actual age. We reviewed published results from studies with relevant information on age at menopause and the risk of hip fracture. Eligible studies that included at least 50 cases were identified through PubMed and the Web of Science, using search terms incorporating “menopause” and “fracture,” through searches for articles citing a well known article on menopause and hip fracture [9], and through hand searches of reference lists of identified articles. Results were collated and, where possible, were summarised as the adjusted RR and 95% CI for hip fracture in women aged <45 y at menopause, compared to women aged 45 y or older at menopause. A total of 12 eligible studies were identified, varying in size from 56 [8] to 2,086 [13] hip fracture cases [6],[8]–[18]. Studies differed in their adjustment for age, socioeconomic factors, use of hormone replacement therapy, smoking, reproductive factors, body mass index, physical activity, and mental status, and only two studies adjusted for comorbidity [6],[10]. Among the studies with relevant information, the estimated RR for hip fracture in women with menopause aged <45 y versus 45 y or older ranged from 1.02 to 1.30 [9],[10],[13],[14], with no studies reporting a significant difference. These point estimates are consistent with the findings of the current study.

Data on bone mineral density suggest that once women are postmenopausal, the age at which they become postmenopausal has only a small effect, which is in keeping with our and other findings regarding the effect of age at menopause and hip fracture incidence. Previous studies have shown that bone mineral density falls most rapidly in the immediate perimenopausal period and less rapidly postmenopausally [2]–[4], with the rate of bone loss declining within around 3 y after menopause [38]. In general it appears that bone mineral density may be reduced in women with early versus late menopause only in the years immediately following menopause [39],[40], but that any difference diminishes with increasing age and is nonexistent or small after the age of 65 y [3],[7],[41]–[43]. Our findings on the incidence of hip fracture in relation to menopause are generally in line with the observed effects of menopause on bone mineral density, as we found about a 2-fold difference in hip fracture incidence between premenopausal and postmenopausal women aged 50–54 y (and there are large differences in bone mineral density between such women), but little or no effect of age at menopause on hip fracture risk in postmenopausal women (and age at menopause appears to have only a weak effect, if any, on long-term bone mineral density in postmenopausal women).

Virtually all hip fractures lead to hospitalisation and occurrences of a first hip fracture in a defined time period are captured well using NHS hospital admission data [44]. Furthermore, the limited data available indicate a relatively high level of accuracy of ICD coding of hip fractures or related diagnoses from hospital discharge data [45]–[47]. All participants are registered with the NHS and were recruited from NHS screening services. Users of such services are likely to use other NHS health services, including NHS hospitals. Use of private hospitals in the UK is limited [48], and many of these admissions are also included in the NHS hospital admission data. Ascertainment of hip fractures occurring in Million Women Study participants using routine NHS hospital data should therefore be virtually complete.

This study has a number of strengths. It is the only study to date, to our knowledge, to combine prospectively gathered data on menopause, including data on premenopausal and perimenopausal women, with complete follow-up for incident hip fractures and adjustment for a wide range of potential confounding factors, including smoking, parity, and comorbidity. Analyses were restricted to women who had never used hormone replacement therapy, to avoid biases resulting from the close relationships between hormone replacement therapy, menopausal status [25], age at menopause [25], and hip fracture [26]. Since serum measures relating to menopausal status, such as serum follicle-stimulating hormone, were not available for study participants, classification of menopausal status relied on self-reported information. Women were considered perimenopausal if they reported irregular menses attributed to menopause, which means that a proportion of women who had regular periods right up until menopause will have been classified as premenopausal during this time. However, the follow-up data on perimenopausal women indicate that such misclassification is likely to be minimal. The inclusion of a small number of perimenopausal women in with premenopausal women may have diluted the effect of menopausal status on hip fracture, yielding more conservative results. Data on other aspects of menopause and potential confounding factors in this study are also self reported. Since all exposure data are prospective, any misclassification should not be biased by the outcome but would tend to dilute the magnitude of any effect. Although Million Women Study participants have been shown to be similar to the general population [49], they are sampled from women attending breast cancer screening in the UK and this should be borne in mind when interpreting study results. While some subgroup comparisons had small numbers of events in each category (Table 3), this is still one of the largest prospective studies to investigate this issue.

What are the clinical implications of these findings? The findings suggest that reduced estrogen around the time of menopause leads to decreased bone mineral density, which in turn increases the risk of hip fracture, relative to premenopausal women. However, hip fracture is rare at the age when most women go through the menopause and increases about 7-fold from age 50–54 to age 70–74. Our findings show that age is far more important than factors relating to menopause in determining the risk of hip fracture. Hence, clinical decisions around hip fracture prevention should be based on age, and age-related factors, such as frailty, low body-mass-index, sensory impairment, and comorbidity, rather than on age at menopause. Factors which increase the risk of falling are particularly important [50]. The evidence suggests that, later in life, women with an early menopause can be reassured that their risk of hip fracture does not differ markedly from that of comparable peers experiencing a later menopause.

Hip fracture becomes an important health issue as women age and is a significant cause of morbidity and mortality from around the age of 70 y onwards—almost one in every 100 never-users of hormone replacement therapy aged 70–74 y in this cohort were admitted to hospital with a hip fracture over a 5-y period; whereas the figure is about seven times lower in women aged 50–54 y. Our findings show that at around the time of menopause, when hip fractures are relatively uncommon, postmenopausal women have a higher incidence of such fractures than premenopausal women. However, after the menopause the incidence of hip fracture increases rapidly with age and fracture rates are determined far more by women's actual ages than by factors relating to the menopause, including the type of menopause and women's ages at menopause.

## Abbreviations

CI: confidence interval
RR: relative risk
